# Effects of a passive upper-body exoskeleton on whole-body kinematics, leg muscle activity, and discomfort during a carrying task

**DOI:** 10.1371/journal.pone.0304606

**Published:** 2024-07-11

**Authors:** Gabriela Garcia, Paul Gonzalo Arauz, Isabel Alvarez, Nicolas Encalada, Shirley Vega, Marco Baldo, Bernard J. Martin

**Affiliations:** 1 Departamento de Ingeniería Industrial, Colegio de Ciencias e Ingenierías, Universidad San Francisco de Quito USFQ, Quito, Ecuador; 2 Department of Orthopaedics, Renaissance School of Medicine, Stony Brook University, New York, NY, United States of America; 3 Department of Industrial and Operations Engineering, University of Michigan, Ann Arbor, Michigan, United States of America; Kennedy Krieger Institute/Johns Hopkins University School of Medicine, UNITED STATES OF AMERICA

## Abstract

**Objective:**

To compare whole-body kinematics, leg muscle activity, and discomfort while performing a 10-min carrying task with and without a passive upper-body exoskeleton (CarrySuit^Ⓡ^), for both males and females.

**Background:**

Diverse commercial passive exoskeletons have appeared on the market claiming to assist lifting or carrying task. However, evidence of their impact on kinematics, muscle activity, and discomfort while performing these tasks are necessary to determine their benefits and/or limitations.

**Method:**

Sixteen females and fourteen males carried a 15kg load with and without a passive exoskeleton during 10-min over a round trip route, in two non-consecutive days. Whole-body kinematics and leg muscle activity were evaluated for each condition. In addition, leg discomfort ratings were quantified before and immediately after the task.

**Results:**

The gastrocnemius and vastus lateralis muscle activity remained constant over the task with the exoskeleton. Without the exoskeleton a small decrease of gastrocnemius median activation was observed regardless of sex, and a small increase in static vastus lateralis activation was observed only for females. Several differences in sagittal, frontal, and transverse movements’ ranges of motion were found between conditions and over the task. With the exoskeleton, ROM in the sagittal plane increased over time for the right ankle and pelvis for both sexes, and knees for males only. Thorax ROMs in the three planes were higher for females only when using the exoskeleton. Leg discomfort was lower with the exoskeleton than without.

**Conclusion:**

The results revealed a positive impact on range of motion, leg muscle activity, and discomfort of the tested exoskeleton.

## Introduction

Exoskeletons have recently drawn increased attention in different jobs due to their commercial availability and adoption interest for industrial or agricultural tasks [1]. Passive exoskeletons, which commonly use elastic components and springs [1] to support the user’s movement, are generally low-cost when compared to active/motorized ones [2], making them more attractive for occupational settings. The majority of passive exoskeletons are designed to support the lower limbs when working while standing, the upper limbs when working with raised arms, and the back for lifting and forward bending operations [3,4]. In addition to lifting, many manual material handling tasks requires the operators to carry and walk with heavy objects. In the US and the European Union, more than 30% of workers have reported being exposed to carrying and transporting heavy loads at their jobs [5]. Several musculoskeletal disorders, such as at the knee, hip, back, neck, and arms, have been linked to carrying and moving large weights [6–8], highlighting the need for interventions for this activity. However, most passive exoskeletons studies have focused on the lifting part of the task [1,9] and less on carrying and walking with heavy objects [10].

Some studies have evaluated exoskeletons carrying tasks through perception of discomfort and performance metrics [11,12]; however, fewer studies have incorporated physiological measures such as muscles activation and joint range of motion [13,14]. In the present study, we focus on the CarrySuit^Ⓡ^, a passive commercially available upper-body exoskeleton released by Auxivo AG (Schwerzenbach, Switzerland). This particular exoskeleton stands out due to its exclusive focus on aiding users in carrying and holding heavy loads, unlike many other passive exoskeletons that target movements like forward bending or other ranges of mobility assistance tasks. To our knowledge, few studies have scientifically evaluated the CarrySuit^Ⓡ^. A recent publication by our team evaluated erector spinae muscle fatigue, upper limb muscle activity, body areas discomfort, and heart rate during a 10-min carrying task with and without the CarrySuit^Ⓡ^ [10]. Other authors performed a single-session evaluation of the CarrySuit^Ⓡ^ through shoulder, neck, and lower back muscle activation during 2-minute load carrying on a treadmill [15]. However, neither kinematics nor muscle activity of the legs was incorporated in the mentioned studies.

Some studies have expressed concerns regarding the increase of leg muscle fatigue with passive industrial exoskeletons [1]. While these exoskeletons aim to alleviate physical strain, they may inadvertently lead to higher leg muscle fatigue levels due to altered biomechanics or improper redistribution of load. By conducting detailed analyses of muscle activation patterns, commonly quantified by the amplitude of the root-mean-square of electromyographic signals [1,3], we can assess whether the CarrySuit^Ⓡ^ effectively redistributes loads without imposing excessive strain on leg muscles. Additionally, issues such as hindering proper posture and movement have been reported in the literature [4,16]. Therefore, examining whole-body kinematics enables us to identify any potential limitations in the range of motion induced by the CarrySuit^Ⓡ^. These concerns underscore the importance of evaluating the biomechanical effects of exoskeletons on users’ leg muscle activity and overall body kinematics. Furthermore, understanding the effects of the CarrySuit^Ⓡ^ on leg muscle activity and whole-body kinematics will complement the validated benefits of previous studies; see [10,15]. Previous studies have highlighted that electromyography and range of motion metrics are relevant objective variables in ergonomic evaluation of exoskeletons [17].

The goal of the present study was to evaluate the influence of using a passive exoskeleton (CarrySuit^Ⓡ^) on muscular activity of the legs, discomfort, and whole-body kinematics during carrying and moving a 15 kg load, considering the influence of sex. The following research questions guided this study:

- Does the gastrocnemius and vastus lateralis muscular activity change over time with or without the CarrySuit^Ⓡ^ during 10 min of carrying and moving a 15kg load?
- Does wearing the CarrySuit^Ⓡ^ influence whole-body kinematics and leg discomfort during the carrying task when compared to not wearing the exoskeleton?
- Is sex an important factor to consider in the present analysis?

## Methods

### Participants

Sixteen females and fourteen males with no previous experience with exoskeletons volunteered to participate in the study. All participants claimed to be free from any musculoskeletal disorders or symptoms, pregnancy, and/or physical or neurological conditions that hinder them from carrying a 15kg load or wearing the tested passive exoskeleton. Demographic data of the participants are as follows: 21.19 **±** 1.6 years of age, 160.16 **±** 4.2 cm stature, and 56.58 **±** 7.24 kg weight for females, and 20.93 **±** 1.49 years of age, 173.39 **±** 6.01 cm stature, and 74.55 **±** 8.8 kg weight for males. Three participants reported being left-handed. All participants signed a written informed consent prior to the experimental sessions. This study was approved by the Ethics Committee of the Universidad San Francisco de Quito (#2021-145M) and complied with the tenets of the Declaration of Helsinki. Participants were recruited from January 13, 2022, until August 15, 2022.

### Passive exoskeleton

**Fig 1:**
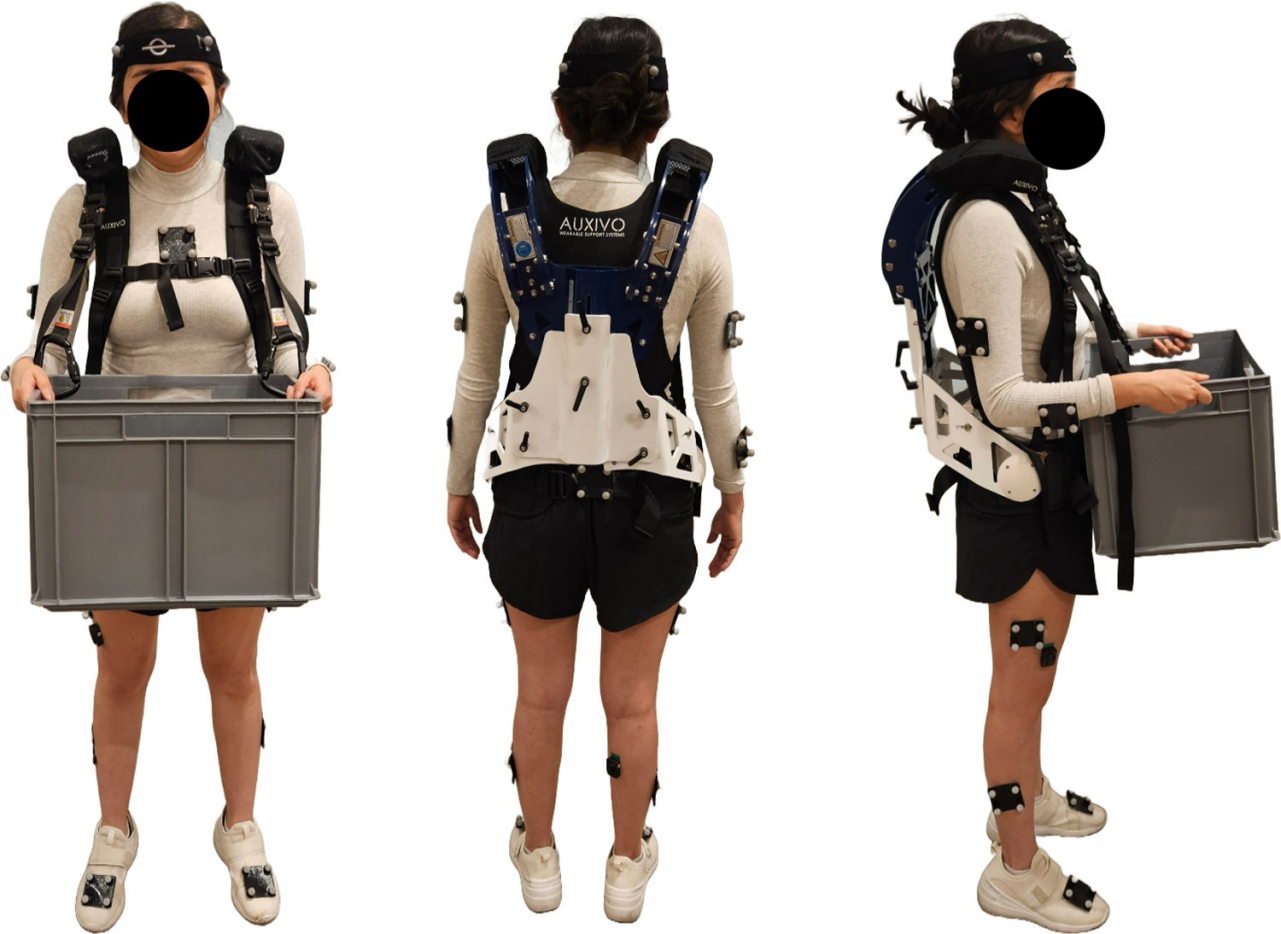
Participant wearing Auxivo CarrySuit^Ⓡ^, motion capture markers and EMG sensors.

In this study, we evaluated the passive upper-body exoskeleton Auxivo CarrySuit^Ⓡ^ v1.0, commercially released in 2021 by Auxivo AG (Schwerzenbach, Switzerland). The CarrySuit^Ⓡ^ was built with the objective of reducing the workload of the upper limbs and back when performing carrying tasks with heavy loads [18]. This exoskeleton has a rigid adjustable frame that extends from the hip to the shoulders. Both the trunk length and hip width are adjustable to match the user’s anthropometry. All metallic contact areas and textile straps are padded, similar to a backpack that connects the hips, chest, and shoulders. Two straps with carabiners hang from the frontal frame to connect external weights (Fig 1). The exoskeleton was designed to support a maximum payload of 50 kg, and it weighs 5.6 kg [18].

### Experimental design and protocol

A detailed description has been presented in a preceding publication [10]. Briefly, in a laboratory setting, participants performed a treatment (with exoskeleton) and control (without exoskeleton) carrying task on two nonconsecutive days assigned in a random order. These days were separated by a minimum of 2 and a maximum of 5 days. Demographic data were recorded only on the first day. For both days, subjective evaluation was followed by motion capture markers and EMG sensors placements. On the treatment day, the exoskeleton was then fitted to each participant according to the manufacturer guidelines and the participant’s comfort. Subsequently, the carrying task was explained and demonstrated to the participant. It consisted of moving a 15kg box back and forward from one table to another. The tables, 3 meters apart, were adjusted to the participant’s elbow height. Instructions required the participants to place the box on the first table for one second before carrying it to the other and repeating. A carrying lap included picking up and returning the box to the first table. To become familiar with the task, 1-minute warm-up laps, with a 5kg load, were performed with or without the exoskeleton depending on the condition tested on the day. Afterwards, the 10-minute carrying task began, during which EMG and motion capture data were recorded every two minutes (T0, T1, T2, T3, T4, T5) for a full carrying lap. All participants were asked to perform as many laps as possible within the 10-minute task period. The number of laps did not differ significantly between conditions, as reported in our previous study [10]. At the end of the carrying task, the subjective evaluation of discomfort was recorded again. Overall, each experimental day took about 1 hour per participant. All participants were instructed to minimize high levels of physical exertion 24 hours before the experimental days.

### Instrumentation and outcome measurements

#### EMG

Gastrocnemius medialis and vastus lateralis muscle activity of the dominant leg were sampled at 1260 Hz with two Avanti EMGs bipolar, surface, and wireless sensors (Delsys Inc, Boston, MA). The sensor areas were shaved, if necessary, and cleaned using abrasive gel (Skin Prep Gel, Nuprep®, Aurora, USA) before sensor placement. Sensors were positioned following SENIAM recommendations [19,20]. The location was temporarily marked to ensure the same position on the second experimental day.

EMG data were processed using a custom script in MATLAB (The MathWorks Inc., Natick, MA, USA). All signals were detrended and filtered with a 4^th^-order Butterworth bandpass filter (30-300Hz). Then, each signal was converted into the frequency domain with Fast Fourier Transform to visually evaluate the data quality. A full-wave rectification was then applied to obtain the absolute value of the filtered signal for normalization purposes. RMS of the EMGs was obtained with a 250ms moving window with 50% overlap. For normalization, the mean-task method was used [21], where the mean activation value obtained during the first 30 seconds of the carrying task was used for each experimental day. Finally, to describe the distribution of muscle activity during the task, the RMS EMG 10^th^, 50^th^, and 90^th^ percentiles (P_10_, P_50_, P_90_) were computed [10,22,23]. These percentiles correspond to the static, mean, and peak muscular activity, respectively [24,25].

#### Kinematics

Whole-body kinematics were obtained from a 10-camera motion capture system (Vicon MX, Oxford, UK) with a sampling frequency of 100 Hz. Based on previous investigations [26–28], a marker model comprising 52 reflective spherical markers (ø 10 mm) was utilized to derive the whole-body kinematics. Clusters of 4 markers were carefully attached to the head, thorax, pelvis, humerus, radius, femur, tibia, and foot (Fig 1) to define the local coordinate system of each segment according to standard recommendations [29]. The 3D joint angles between head and trunk, pelvis and trunk, as well as angles of the shoulder (between humerus and thorax), elbow (between humerus and radius), hip (between pelvis and femur), knee (between femur and tibia), and ankle (between foot and tibia) were calculated. Segment and joint angles were determined using a Cardan angle sequence [30]. Data were exported and processed in MATLAB (MathWorks, Inc., Natick, MA) using a custom program.

Segment and joint angles for a relaxed standing position were used as the neutral zero reference. The angular data were divided into separate strides, and using 1% sample steps, a time-normalized waveform (0–100%) of the typical gait cycle was created [26,27,31,32]. Normalized strides were specified to begin with the first foot contact (0%) and terminate (100%) with the next foot contact. The complete gait cycle of the right leg was selected for each of the two-minute recordings (T0—T5) for analysis. To illustrate the referenced angles, an example of the kinematic curves in three-dimensional planes for the five time periods, both with and without the exoskeleton, of one subject is presented in the supporting information S1 Fig. Specifically, the supporting information depicts the left ankle, left knee, left hip; right ankle, right knee, right hip left and right elbow, pelvis, thorax, and neck.

#### Subjective evaluation

Perception of discomfort ratings of the upper legs/hip, knees, lower leg, ankles, and feet were obtained through an adaptation of the Nordic questionnaire [33], as in [10,34–36]. A human body diagram with 10 cm visual analogue scales for each body part was presented before and after the carrying task. The scales corresponded to 10cm segments, where “no discomfort” corresponded to the extreme left (0) and “extreme discomfort” to the extreme right (10). Participants placed vertical marks over the scales indicating their discomfort level. The results are presented in millimeters.

### Data analysis

All statistical analyses were performed in SAS Studio (SAS Institute Inc.) with α = .05. Mixed models with a variance-components covariance structure with residual maximum likelihood estimation were used to analyze the following outcome variables: P_10_, P_50_, and P_90_ RMS EMG, and body segments’ range of motion (ROM). For the EMG statistical models, participants were considered as random effects, and measurement time and sex were considered as fixed effects. Independent models were run for each experimental condition (with [EXO] or without the exoskeleton [NOEXO]) since normalization was performed to the mean muscle activation recorded at T0 on the corresponding day. For ROM statistical models, “condition” was also included as a fixed effect. Least square means with Tukey-Kramer adjustment of p-values were used for post-hoc analysis. Means (M) and standard errors (SE) are described in the results. Partial eta-squared pseudo-effect size (η_p_^2^) was obtained with a method for mixed models in SAS Studio [37]. Small (η_p_^2^ = .01), medium (η_p_^2^ = .06), and large effects (η_p_^2^ = .14) were used for the interpretation of η_p_^2^ [38]. Discomfort ratings did not fulfill the normality assumption; thus, a Friedman nonparametric two-way analysis of variance by ranks test was applied.

## Results

### Gastrocnemius medialis RMS EMG

**Fig 2:**
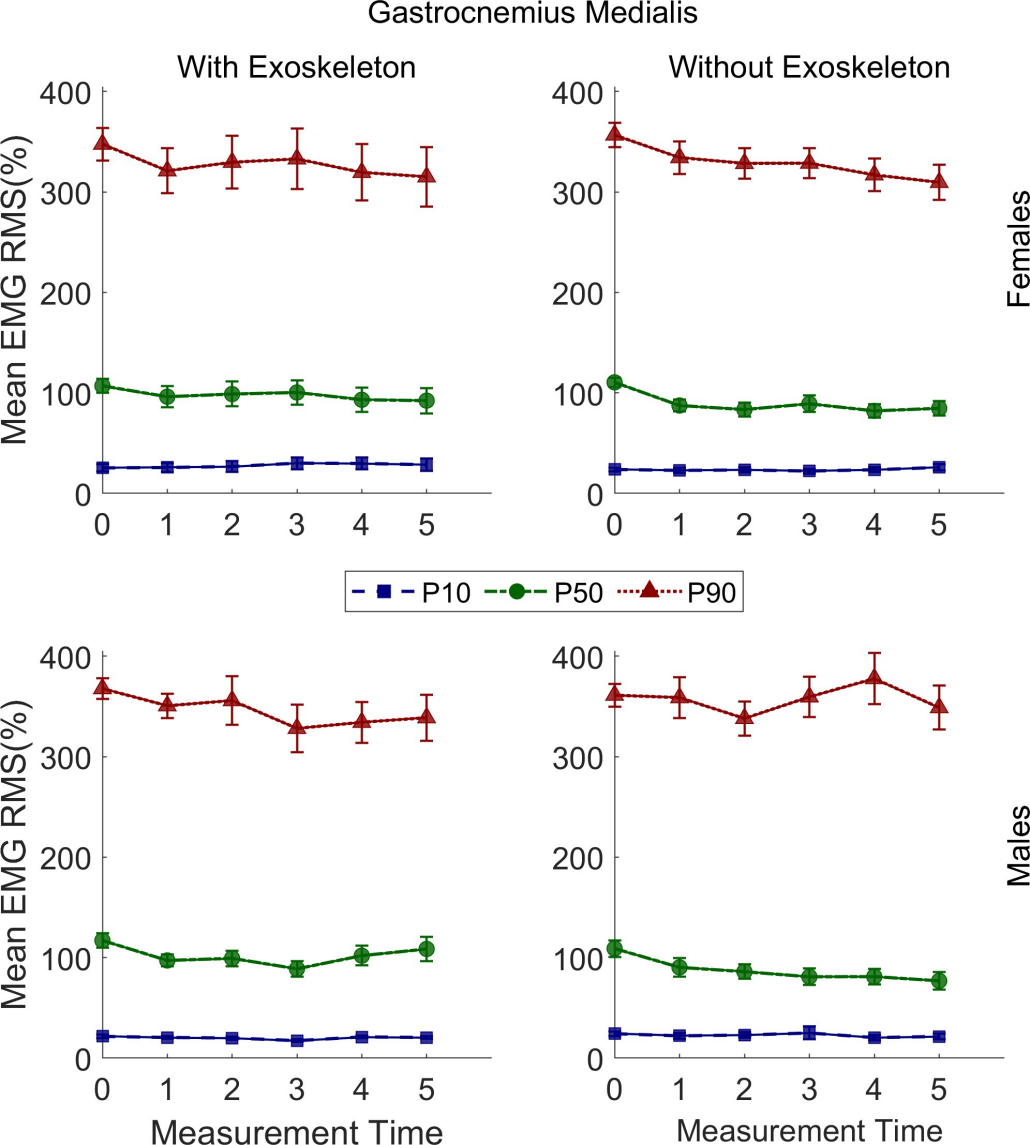
Gastrocnemius RMS EMG 10^th^, 50^th^ and 90^th^ percentiles of males and females under both conditions. Vertical bars indicate standard errors.

The RMS EMG P_10_, P_50_, and P_90_ in both conditions are presented in Table 1. No significant differences were found in the EXO condition. However, in the NOEXO condition a significant decrease in P_50_ from T0 (M = 109.59%, SE = 5.19%) to T1(M = 88.92%, SE = 5.16%; adj p = .001), which remained constant through T5(M = 81.77%, SE = 5.27%; adj p = < .0001) was found regardless of sex. No significant changes were observed for P_10_ and P_90_ in this condition. These results are illustrated in Fig 2.

**Table 1:** Gastrocnemius medialis RMS EMG percentiles.

		10^th^ percentile (P_10_)			50^th^ percentile (P_50_)			90^th^ percentile (P_90_)		
Condition	Effect	F value	p value	η_p_^2^	F value	p value	η_p_^2^	F value	p value	η_p_^2^
With Exoskeleton	Sex	1.73	0.19	0.01	0.05	0.82	0.0003	0.35	0.55	0.002
	Time	1.69	0.14	0.05	2.01	0.08	0.06	1.41	0.22	0.05
	Sex x Time	1.95	0.09	0.06	0.81	0.54	0.03	0.35	0.88	0.01
Without Exoskeleton	Sex	0.26	0.61	0.001	0.01	0.99	0.00	1.77	0.18	0.009
	Time	0.36	0.87	0.01	8.10	**< .0001** *	0.18	2.20	0.06	0.06
	Sex x Time	1.00	0.42	0.03	0.43	0.82	0.01	0.96	0.44	0.03

Note. Bold font and

* indicate significant values, α = 0.05.

### Vastus lateralis RMS EMG

**Fig 3:**
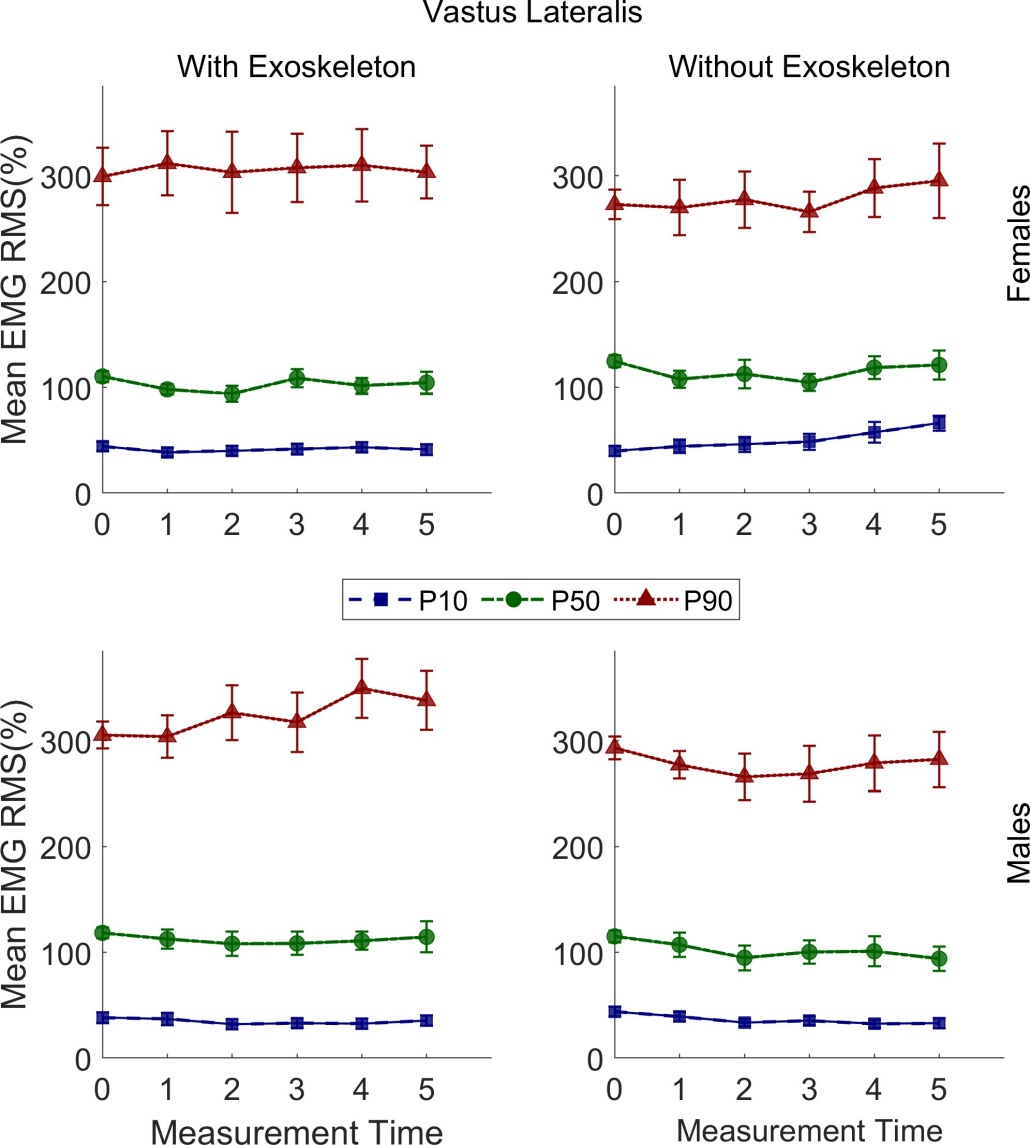
Vastus lateralis RMS EMG 10th, 50th and 90th percentiles of males and females under both conditions. Vertical bars indicate standard errors.

RMS EMG P_10_, P_50_, and P_90_ in both conditions are presented in Table 2. No significant differences were found in the EXO condition. However, in the NOEXO condition post hoc comparisons showed a significant increase in P_10_ for females at T5 (*M* = 62.57%, SE = 5.56%) when compared to T0 (*M* = 40.35%, SE = 4.87%; adj p = .01) but not for males. No significant changes were observed for P_50_ and P_90_ in this condition. These results are illustrated in Fig 3.

**Table 2:** Vastus lateralis RMS EMG percentiles.

		10^th^ percentile (P_10_)			50^th^ percentile (P_50_)			90^th^ percentile (P_90_)		
Condition	Effect	F value	p value	η_p_^2^	F value	p value	η_p_^2^	F value	p value	η_p_^2^
With Exoskeleton	Sex	0.73	0.39	0.01	0.77	0.38	0.01	0.17	0.67	0.001
	Time	0.88	0.50	0.03	1.28	0.27	0.05	0.67	0.64	0.03
	Sex x Time	0.72	0.61	0.03	0.40	0.84	0.02	0.57	0.57	0.03
Without Exoskeleton	Sex	8.24	**0.005** *	0.04	0.91	0.34	0.01	1.84	0.17	0.01
	Time	0.85	0.51	0.02	2.03	0.08	0.05	1.63	0.15	0.04
	Sex x Time	3.40	**0.007** *	0.09	1.81	0.11	0.05	2.03	0.08	0.05

Note. Bold font and

* indicate significant values, α = 0.05.

### Sagittal plane movements range of motion

**Fig 4:**
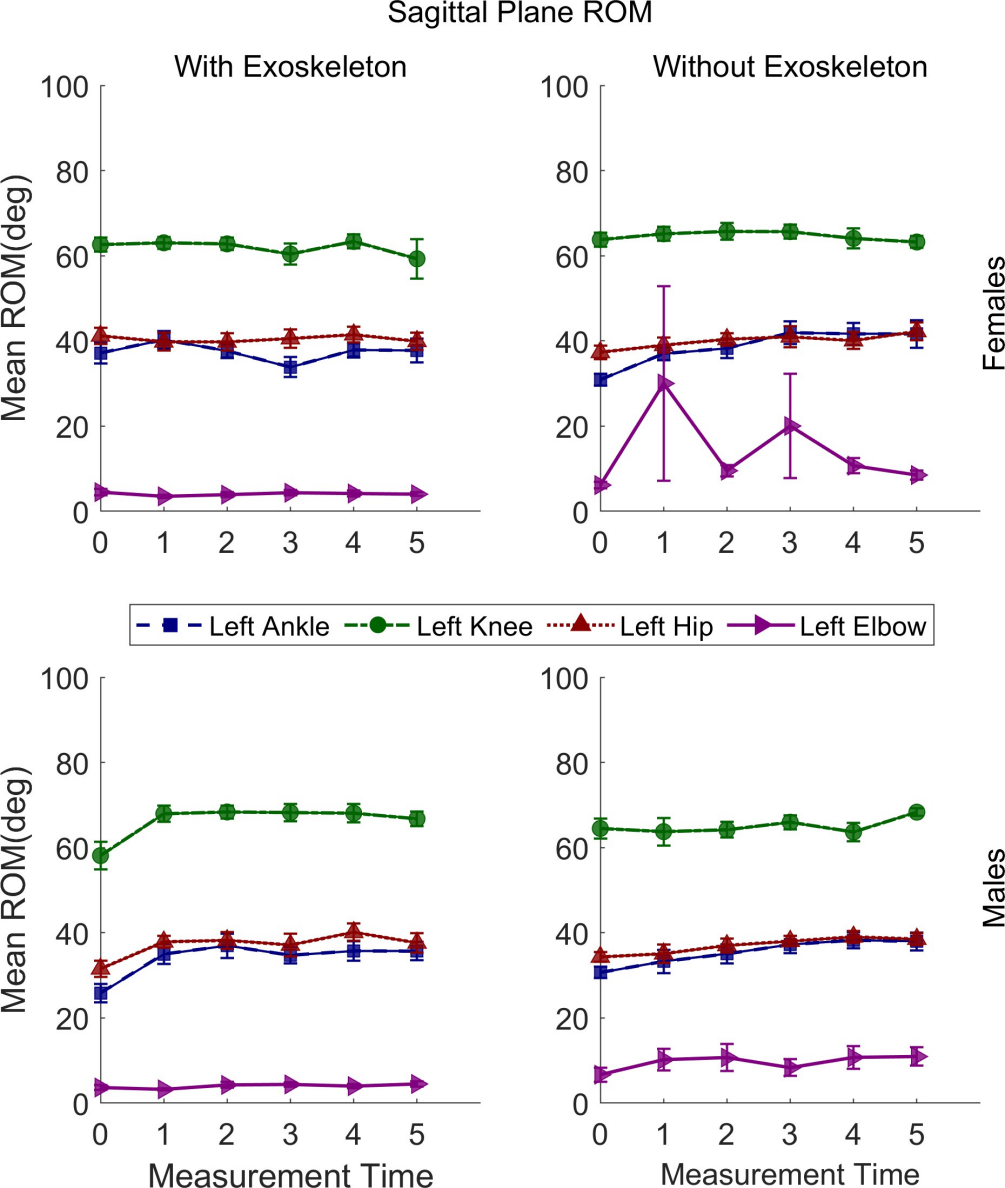
Sagittal plane range of motion means for the left ankle, left knee, left hip, and left elbow of males and females under both conditions. Vertical bars indicate standard errors.

**Fig 5:**
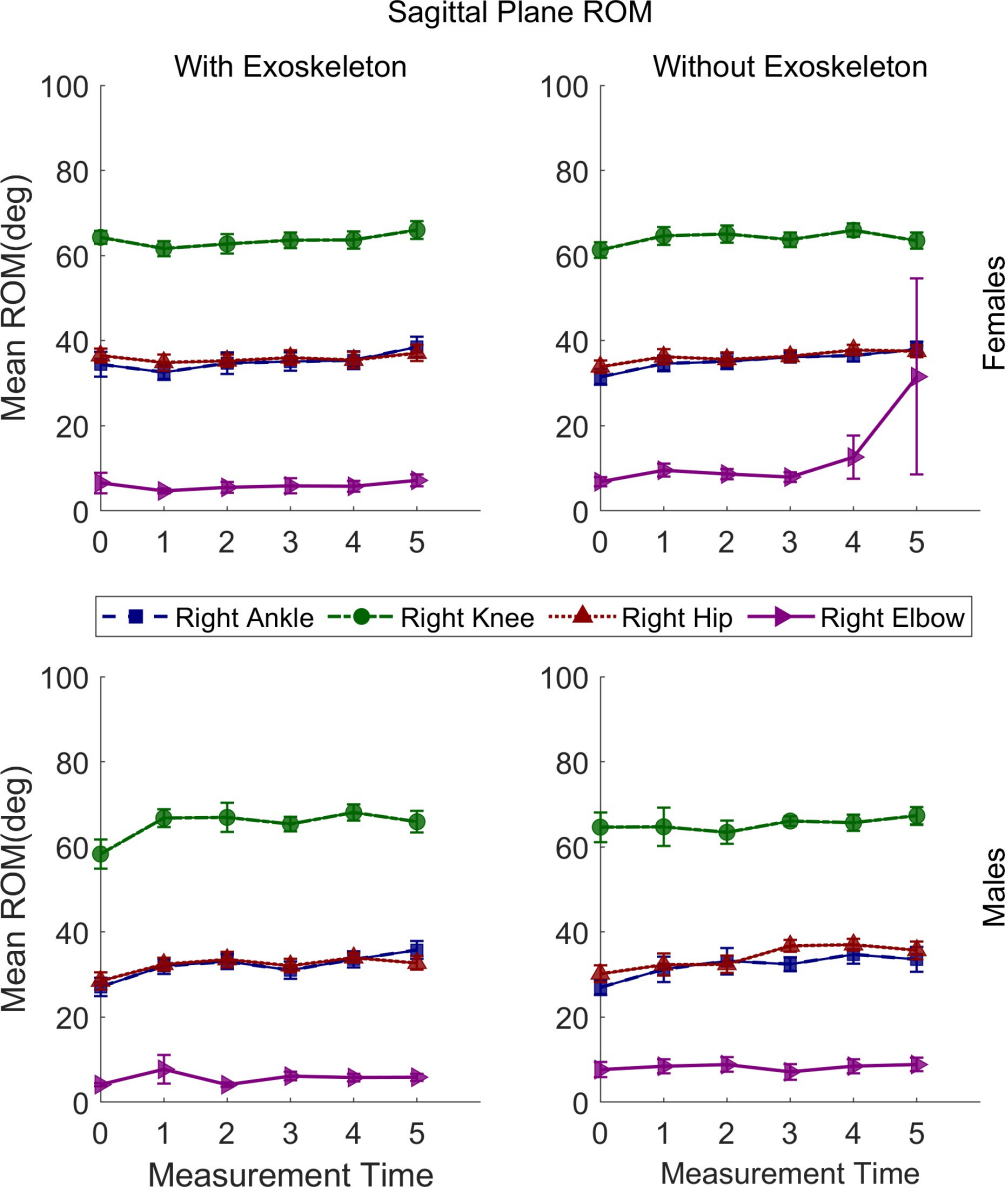
Sagittal plane range of motion means for the right ankle, right knee, right hip, and right elbow of males and females under both conditions. Vertical bars indicate standard errors.

**Fig 6:**
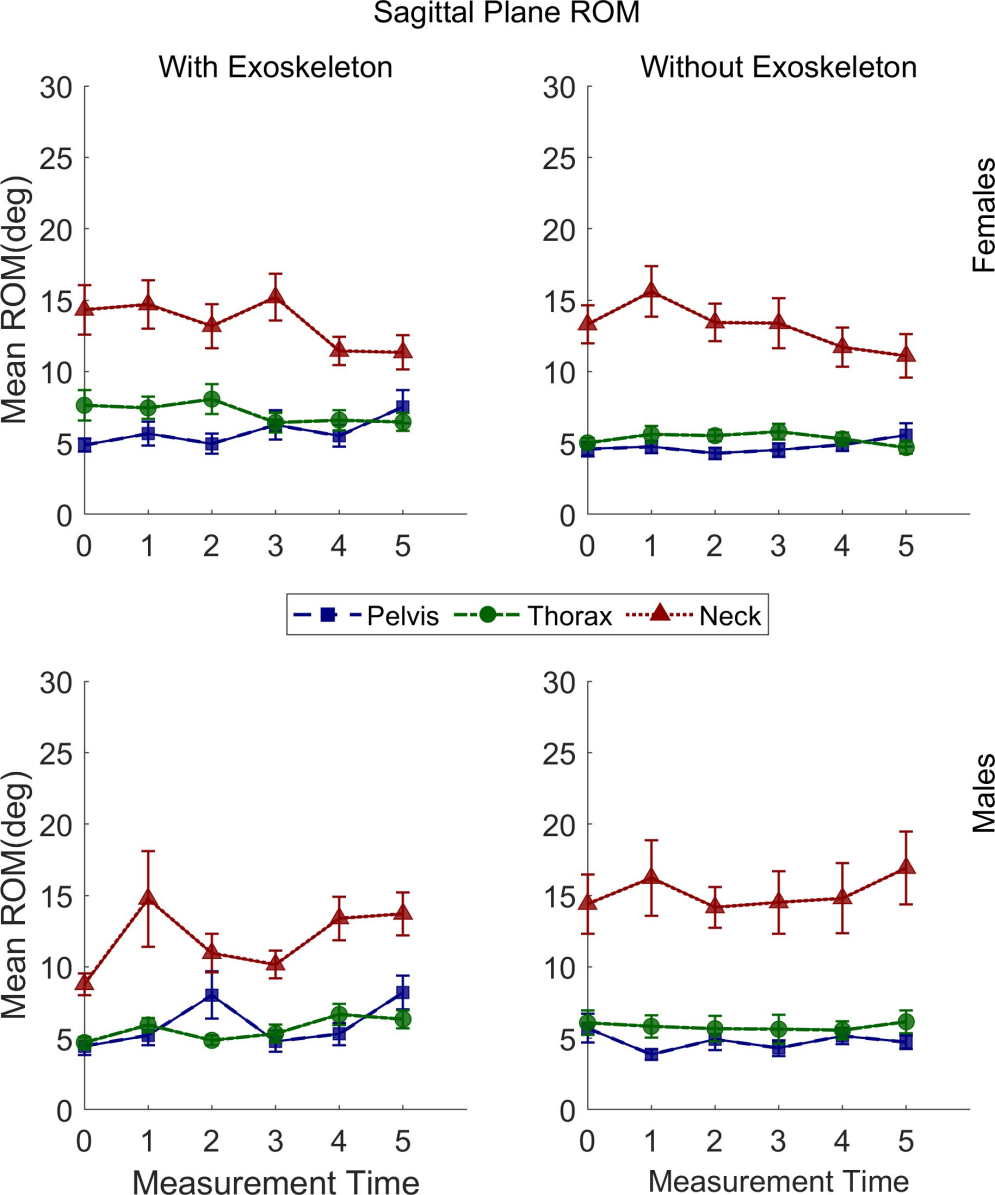
Sagittal plane range of motion means for the pelvis, thorax, and neck of males and females under both conditions. Vertical bars indicate standard errors.

The statistical results of sagittal plane movements’ ROM are presented in Table 3. Some interactions and main effects were significant for all joints except the shoulders. The relevant post hoc comparisons are described in the following sections and illustrated in Figs 4–6.

**Table 3:** Statistical analysis of the ranges of motion for movements in the sagittal plane.

Joint	Effect						
	P values (η_p_^2^)						
	Condition	Time	Sex	Sex*Time	Condition*Time	Sex*Condition	Sex*Condition*Time
Left Ankle	0.07 (0.01)	**< .0001** * **(0.12)**	0.15(0.006)	0.31(0.02)	**0.01** * **(0.04)**	0.83(0.0001)	**0.01** * **(0.04)**
Right Ankle	0.92(0.0001)	**< .0001** * **(0.11)**	0.17(0.005)	0.31(0.02)	0.66(0.01)	0.83(0.0001)	0.86(0.005)
Left Knee	0.32 (0.003)	0.18(0.02)	0.14(0.006)	0.06(0.03)	0.19(0.02)	**0.01** * **(0.02)**	**0.02** * **(0.02)**
Right Knee	0.76(0.0002)	0.06(0.03)	0.43(0.002)	0.65(0.009)	0.95(0.003)	0.85(0.00001)	**0.03** * **(0.04)**
Left Hip	0.65(0.0001)	**0.0003** * **(0.06)**	0.07(0.01)	0.14(0.02)	0.56(0.01)	0.74(0.0003)	0.26(0.02)
Right Hip	0.05(0.01)	**0.002** * **(0.05)**	0.05(0.01)	0.22(0.02)	0.42(0.01)	0.19(0.005)	0.42(0.01)
Pelvis	**0.0003** * **(0.04)**	**0.02** * **(0.04)**	0.72(0.0003)	0.17(0.02)	0.05(0.03)	0.77(0.0002)	0.34(0.01)
Thorax	**0.002** * **(0.03)**	0.97(0.003)	0.29(0.003)	0.22(0.02)	0.89(0.005)	**0.0002** * **(0.04)**	0.27(0.02)
Left Shoulder	0.18(0.005)	0.41(0.01)	0.76(0.0003)	0.25(0.02)	0.73(0.008)	0.54(0.001)	0.82(0.006)
Right Shoulder	0.68(0.0005)	0.07(0.03)	0.15(0.006)	0.31(0.02)	0.52(0.01)	0.07(0.01)	0.28(0.02)
Left Elbow	**0.001** * **(0.03)**	0.73(0.008)	0.38(0.002)	0.71(0.008)	0.63(0.009)	0.36(0.002)	0.73(0.007)
Right Elbow	**0.01** * **(0.02)**	0.32(0.02)	0.37(0.002)	0.45(0.01)	0.48(0.01)	0.27(0.003)	0.51(0.01)
Neck	**0.03** * **(0.02)**	0.24(0.02)	0.76(0.0002)	**0.04** * **(0.03)**	0.99(0.001)	**0.01** * **(0.02)**	0.71(0.008)

Note. Bold font and

* indicate significant values, α = 0.05.

#### Ankles dorsi-plantar flexion ROM

A significant increase in the left ankle dorsi-plantar flexion ROM was found from T0 (M = 30.90°, SE = 2.11) to T3 (M = 42.34°, SE = 2.16; adj p = .01) and remained high through T5 only for females in NEXO but not in EXO. For the right ankle a significant increase was found from T0 (M = 29.92°, SE = 1.28) to T2 (M = 32.52°, SE = 1.28; adj p = .004) and continued to increase through T5 (M = 36.13°, SE = 1.29; adj p = < .0001) for both conditions, regardless of sex.

#### Knees flexion-extension ROM

A significant increase in the left knee flexion-extension ROM was found from T0 (M = 57.96°, SE = 2.21) to T2 (M = 68.60°, SE = 2.29; adj p = .03) and remained high through T5 only for males in EXO but not in NOEXO. Similar for the right knee a significant increase was found fromT0 (M = 58.16°, SE = 2.35) to T4 (M = 68.09°, SE = 2.28; adj p = .04) and remained high through T5 only for males in EXO but not in NOEXO.

#### Hips flexion-extension ROM

A significant increase in the left hip flexion-extension ROM was found from T0 (M = 35.67°, SE = 1.32) to T5 (M = 40.45°, SE = 1.33; adj p = .04) in NOEXO and from T0 (M = 36.21°, SE = 1.32) to T4 (M = 40.79°, SE = 1.30; adj p = .05) and remained high through T5 in EXO, regardless of sex. For the right hip a significant increase was found from T0 (M = 32.03°, SE = 1.19) to T4 (M = 37.42°, SE = 1.17; adj p = .007) in NOEXO but not in EXO, regardless of sex.

#### Elbows flexion-extension ROM

For the left elbow a significantly greater flexion-extension ROM was found in NOEXO (M = 11.87°, SE = 1.80) than in EXO (M = 4.03°, SE = 1.81; adj p = .001), regardless of sex and time. Similarly, for the right elbow a significantly greater FE-ROM was found in NOEXO (M = 10.51°, SE = 1.68) than in EXO (M = 5.75°, SE = 1.68; adj p = .01), regardless of sex and time.

#### Pelvis posterior-anterior tilt ROM

A significant increase in the pelvis posterior-anterior tilt ROM was found from T0 (M = 4.64°, SE = .53) to T5 (M = 7.85°, SE = .54; adj p = .001) in EXO but not in NOEXO regardless of sex.

#### Thorax posterior-anterior tilt ROM

For the thorax a significantly greater posterior-anterior Tilt ROM was found only for females in EXO (M = 7.12°, SE = .36) than NOEXO (M = 5.32°, SE = .37; adj p = < .0001), regardless of time.

#### Neck flexion-extension RO

For the neck a significantly greater ROM was found only for males in NOEXO (M = 15.16°, SE = .94) than in EXO (M = 11.95°, SE = .94; adj p = .008), regardless of time.

### Frontal plane movements range of motion

**Fig 7:**
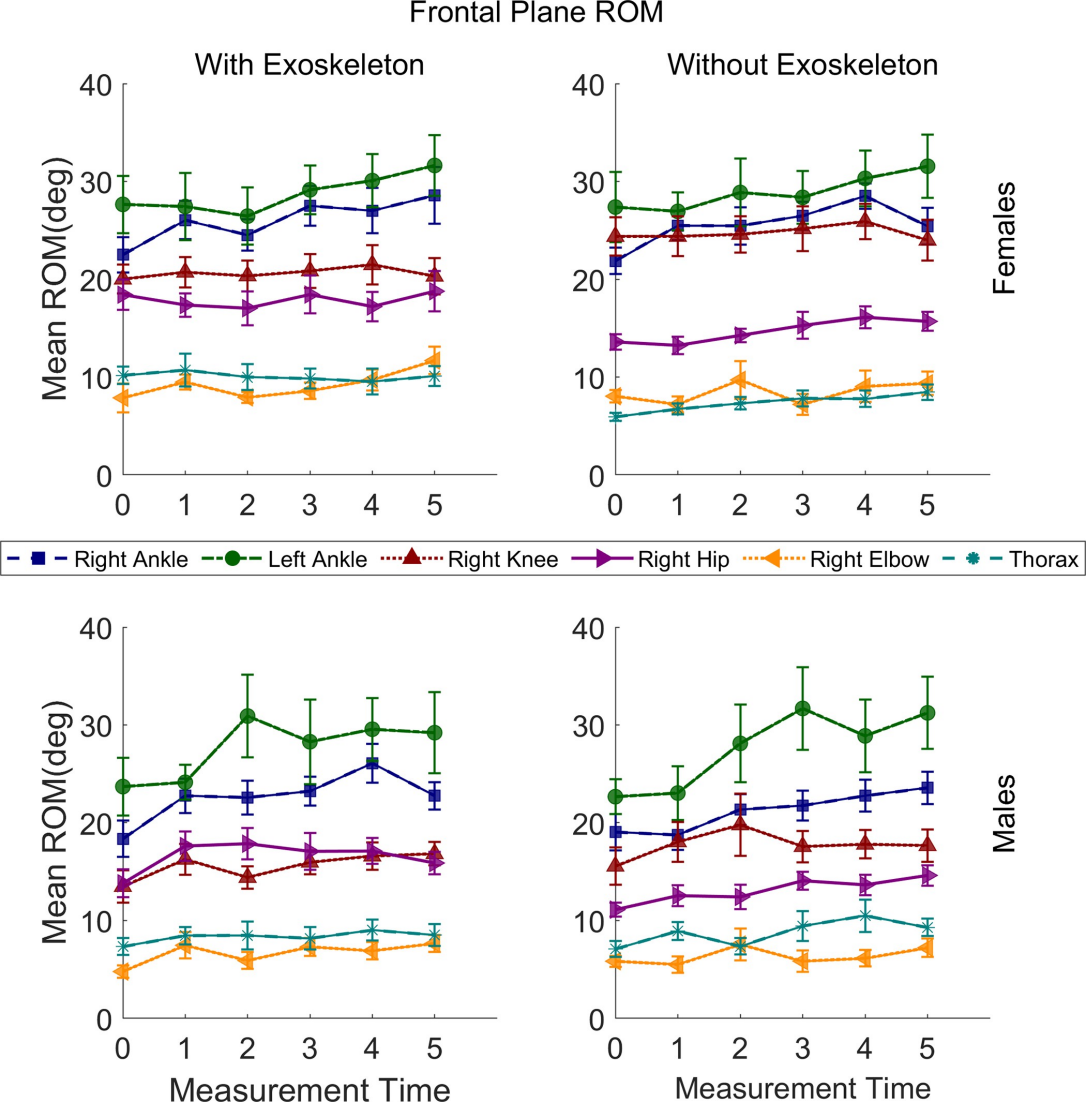
Frontal plane range of motion means for the right ankle, left ankle, right knee, right hip, right elbow, and thorax of males and females under both conditions. Vertical bars indicate standard errors.

The statistical results of frontal plane movements’ ROM are presented in Table 4. Some interactions and main effects were significant for all joints except the pelvis and neck. The relevant post hoc comparisons are described in the following sections, and illustrated in Fig 7; no significant differences were found for the left and right shoulder, left knee, and left elbow.

**Table 4:** Statistical analysis of the ranges of motion for movements in the frontal plane.

Joint	Effect						
	P values (η_p_^2^)						
	Condition	Time	Sex	Sex*Time	Condition*Time	Sex*Condition	Sex*Condition*Time
Left Ankle	0.94(0.00001)	**0.01** * **(0.04)**	0.66(0.0005)	0.48(0.012)	0.99(0.001)	0.92(0.00002)	0.88(0.005)
Right Ankle	0.13(0.006)	**<0.0001** * **(0.08)**	**0.007(0.02)**	0.96(0.002)	0.91(0.004)	0.46(0.001)	0.37(0.01)
Left Knee	0.07(0.02)	0.38(0.01)	**0.01** * **(0.02)**	0.97(0.002)	0.53(0.01)	0.15(0.005)	0.41(0.01)
Right Knee	**<0.0001** * **(0.07)**	0.49(0.01)	**0.0002** * **(0.04)**	0.74(0.008)	0.88(0.005)	0.11(0.007)	0.92(0.004)
Left Hip	0.47(0.001)	**0.002** * **(0.05)**	0.99(0)	0.07(0.03)	0.51(0.01)	0.36(0.002)	0.63(0.009)
Right Hip	**<0.0001** * **(0.12)**	0.09(0.03)	0.13(0.006)	0.36(0.01)	0.59(0.01)	0.74(0.0003)	0.57(0.01)
Pelvis	0.34(0.002)	0.81(0.006)	0.22(0.004)	0.37(0.01)	0.27(0.01)	0.49(0.001)	0.30(0.01)
Thorax	**0.0009** * **(0.03)**	0.06(0.02)	0.87(0.0001)	0.55(0.01)	0.29(0.01)	**<0.0001** * **(0.06)**	0.85(0.005)
Left Shoulder	0.26(0.003)	**0.007** * **(0.04)**	0.83(0.0001)	**0.04** * **(0.03)**	0.85(0.005)	0.95(0.0000)	0.68(0.008)
Right Shoulder	0.16(0.005)	**0.04** * **(0.03)**	0.81(0.0001)	0.99(0.001)	0.16(0.02)	**0.02** * **(0.01)**	0.7(0.008)
Left Elbow	0.33(0.002)	0.74(0.007)	**0.002** * **(0.02)**	0.06(0.02)	0.26(0.01)	0.52(0.001)	0.42(0.01)
Right Elbow	0.14(0.005)	**0.03** * **(0.03)**	**0.01** * **(0.02)**	0.81(0.006)	**0.03** * **(0.03)**	0.54(0.001)	0.97(0.002)
Neck	0.12(0.006)	0.50(0.01)	0.79(0.0001)	0.76(0.007)	0.11(0.02)	0.54(0.001)	0.13(0.02)

Note. Bold font and

* indicate significant values, α = 0.05.

#### Ankles eversion-inversion ROM

A significant increase in the left ankle eversion-inversion ROM was found from T0 (M = 25.34°, SE = 1.81) to T5 (M = 30.96°, SE = 1.83; adj p = .03) for both conditions, regardless of sex. For the right ankle a significant increase was found from T0 (M = 20.57°, SE = .99) to T3 (M = 24.62°, SE = 1.01; adj p = .004) and remained high through T5 for both conditions, regardless of sex.

#### Knees abduction-adduction ROM

For the right knee a significantly greater abduction-adduction ROM was found only for females in NOEXO (M = 24.64°, SE = 1.13) than in EXO (M = 20.52°, SE = 1.13; adj p = < .0001), regardless of time.

#### Hips abduction-adduction ROM

For the left hip no relevant differences were found. For the right hip a significantly greater abduction-adduction ROM was found in EXO (M = 17.18°, SE = .57) than in NOEXO (M = 13.85°, SE = .57; adj p = < .0001), regardless of sex and time.

#### Thorax ipsi-contra lateral tilt ROM

For the thorax a significantly greater ROM was found only for females in EXO (M = 10.05°, SE = .71) than in NOEXO (M = 7.33°, SE = .71; adj p = < .0001), regardless of time.

#### Elbows abduction-adduction ROM

For the right elbow a significant increase was found from T0 (M = 6.37°, SE = .77) to T5 (M = 9.61°, SE = .78; adj p = .02) only in EXO, regardless of sex.

### Transverse plane movements range of motion

**Fig 8:**
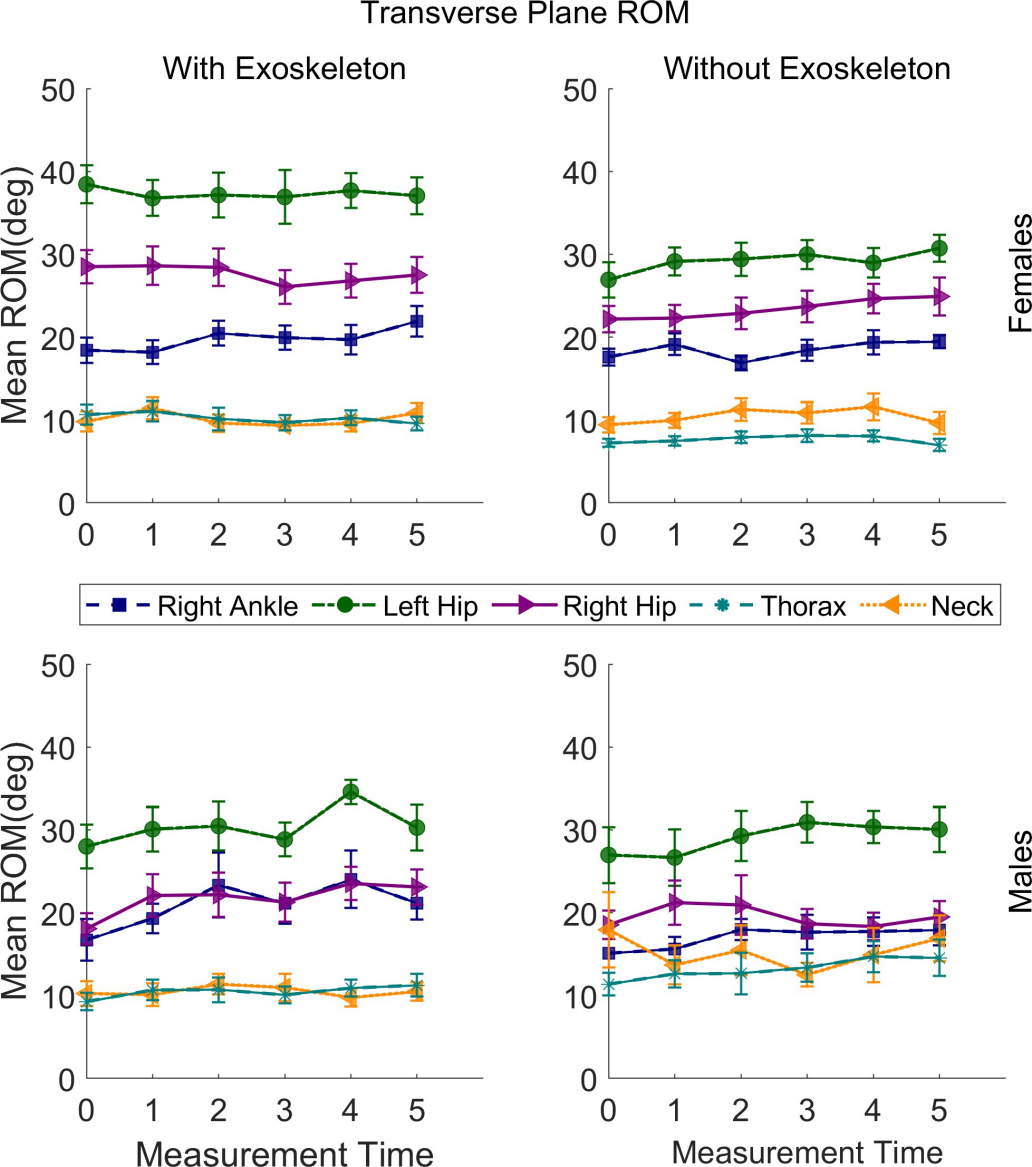
Transverse plane range of motion means for the right ankle, left hip, right hip, thorax, and neck of males and females under both conditions. Vertical bars indicate standard errors.

The statistical results of transverse plane movements’ ROM are presented in Table 5. Some interactions and main effects were significant for all joints except for the right knee, pelvis, right shoulder, and right elbow. The relevant post hoc comparisons are described in the following sections, and illustrated in Fig 8; no significant differences were found for the left shoulder, ankle, knee, and elbow.

**Table 5:** Statistical analysis of the ranges of motion for movements in the transverse plane.

Joint	Effect						
	P values (η_p_^2^)						
	Condition	Time	Sex	Sex*Time	Condition*Time	Sex*Condition	Sex*Condition*Time
Left Ankle	0.33(0.002)	**0.04** * **(0.03)**	0.84(0.0001)	0.53(0.01)	0.15(0.02)	0.38(0.002)	0.78(0.006)
Right Ankle	**<0.0001** * **(0.05)**	**0.01** * **(0.04)**	0.84(0.0001)	0.41(0.01)	0.68(0.008)	**0.03** * **(0.01)**	0.77(0.006)
Left Knee	0.28(0.003)	**0.04** * **(0.03)**	**0.009** * **(0.02)**	0.89(0.004)	0.73(0.007)	0.15(0.005)	0.80(0.006)
Right Knee	0.01*(0.02)	0.07(0.02)	0.0008*(0.03)	0.33(0.01)	0.93(0.003)	0.98(0.000)	0.77(0.007)
Left Hip	**<0.0001** * **(0.09)**	0.41(0.01)	0.11(0.006)	0.72(0.008)	0.57(0.01)	**<0.0001** * **(0.05)**	0.81(0.006)
Right Hip	**<0.0001** * **(0.05)**	0.47(0.01)	0.006(0.02)	0.79(0.006)	0.99(0.001)	0.15(0.01)	0.25(0.01)
Pelvis	0.89(0.0000)	0.22(0.01)	0.98(0.0000)	0.13(0.02)	0.64(0.009)	0.95(0.0000)	0.99(0.001)
Thorax	0.84(0.0001)	0.31(0.01)	**0.02** * **(0.01)**	0.19(0.02)	0.67(0.008)	**<0.0001** * **(0.12)**	0.98(0.001)
Left Shoulder	0.23(0.003)	0.81(0.006)	**0.01** * **(0.02)**	0.15(0.02)	0.64(0.009)	0.60(0.0007)	0.31(0.01)
Right Shoulder	0.30(0.002)	0.26(0.01)	0.06(0.009)	0.74(0.007)	0.69(0.008)	0.53(0.001)	0.52(0.01)
Left Elbow	**0.03** * **(0.01)**	0.71(0.008)	0.27(0.003)	0.64(0.009)	0.61(0.009)	0.24(0.003)	0.70(0.008)
Right Elbow	0.11(0.006)	0.29(0.01)	0.25(0.003)	0.41(0.01)	0.66(0.009)	0.31(0.002)	0.68(0.008)
Neck	**0.0003** * **(0.04)**	0.92(0.003)	**0.008** * **(0.02)**	0.73(0.007)	0.85(0.005)	**0.001** * **(0.03)**	0.52(0.01)

Note. Bold font and

* indicate significant values, α = 0.05.

#### Ankles internal-external rotation ROM

For the right ankle a significantly greater ROM was found in EXO (M = 20.78°, SE = 1.18) than in NOEXO (M = 16.83°, SE = 1.19; adj p = .0002) only for males, regardless of time.

#### Hips internal-external rotation ROM

For the left hip a significantly greater ROM was found in EXO (M = 37.32°, SE = 1.57) than in NOEXO (M = 29.16°, SE = 1.58; adj p = < .0001) only for females, regardless of time. For the right hip a significantly greater IE-ROM was found in EXO (M = 24.61°, SE = .97) than in NOEXO (M = 21.41°, SE = .97; adj p = .0001), regardless of sex and time.

#### Thorax ipsi-contra lateral rotation ROM

For the thorax a significantly greater ROM was found for females in EXO (M = 10.28°, SE = .87) than in NOEXO (M = 7.69°, SE = .87; adj p = < .0001), regardless of time. However, for the males a significantly greater IE-ROM was found in NOEXO (M = 13.20°, SE = .94) than in EXO (M = 10.46°, SE = .94; adj p = < .0001), regardless of time.

#### Neck Axial-Rotation–ROM

For the neck a significantly greater ROM was found in NOEXO (M = 15.29°, SE = .88) than in EXO (M = 10.55°, SE = .88; adj p = < .0001) only for males, regardless of time.

### Subjective evaluation of discomfort

**Fig 9:**
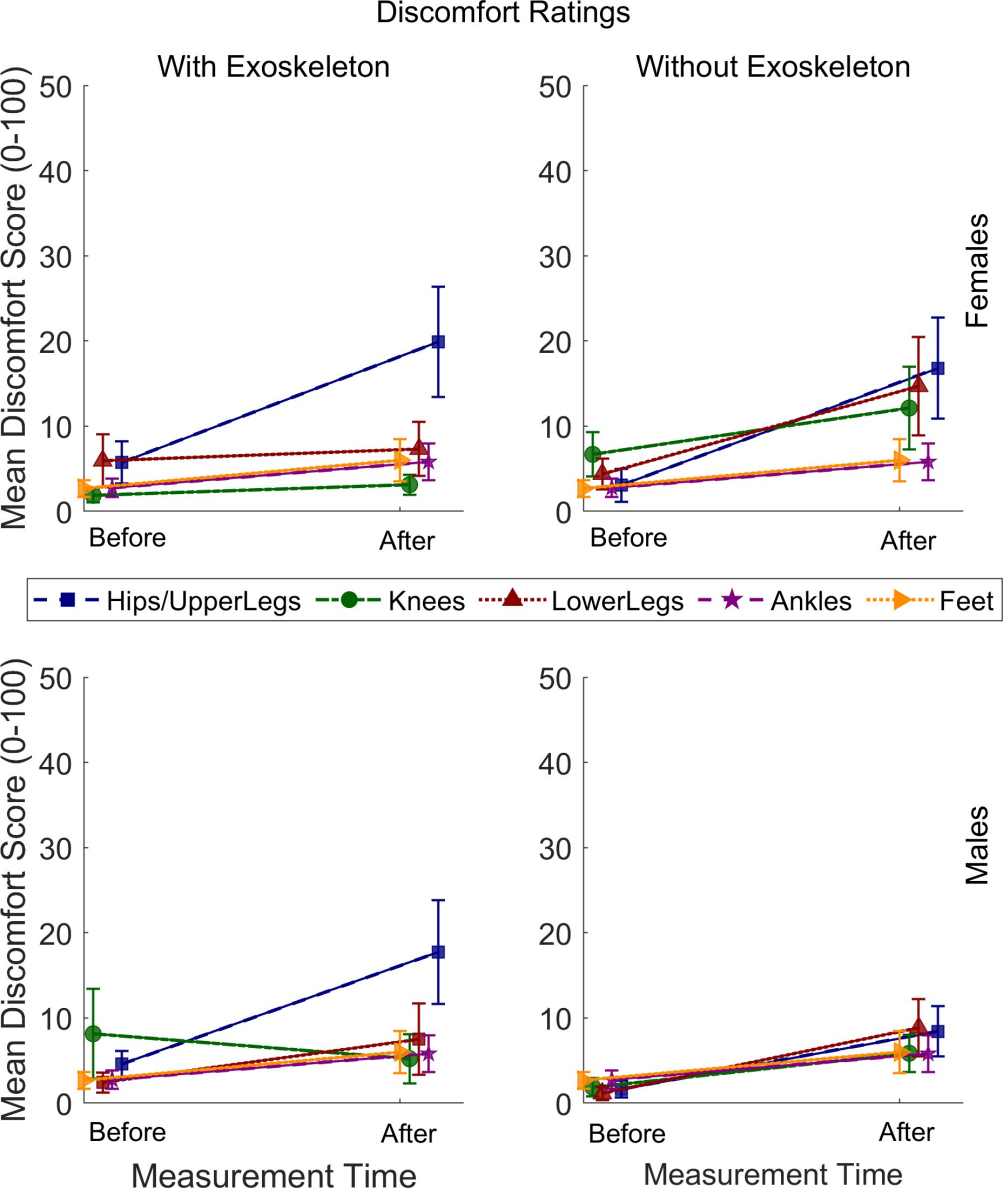
Discomfort ratings of males and females under both conditions. Vertical bars indicate standard errors.

Friedman test comparisons for discomfort rating are shown in Table 6. The comparisons for the ratings before and after the carrying task within each condition are presented on the left side of the table, along with the mean and standard deviation. These results indicate significantly greater discomfort in all tested body areas without the exoskeleton, but only for the hip/upper legs and feet with the exoskeleton, for both females and males (Fig 9). The right side of Table 6 shows the comparison between the exoskeleton and no exoskeleton condition by analyzing the difference (Δ) between ratings reported after and those reported before the carrying task. These results show that discomfort in the knees, lower legs, and ankles was higher without the exoskeleton than with the exoskeleton. No significant differences were found between females and males.

**Table 6:** Discomfort ratings.

	Before vs After Carrying Task								With vs Without Exoskeleton	
	With Exoskeleton				Without Exoskeleton				Δ(After-Before)	
	Before Mean(Std)	After Mean (Std)	F value	p value	Before Mean(Std)	After Mean (Std)	F value	p value	F value	p value
Hip/Upper Legs	5.2(8.19)	18.9(24.1)	4.28	**0.04** *	2.4(6.1)	12.9(19.1)	5.18	**0.03** *	0.33	0.57
Knees	4.8(13.8)	4.1(8.04)	1	0.45	4.4(8.4)	9.2(15.3)	6.59	**0.02** *	9.41	**0.005** *
Lower Legs	4.3(9.66)	7.4(13.9)	2.38	0.13	2.9(5.8)	12(18.8)	21.58	**<0.0001** *	5.8	**0.02** *
Ankles	2.4(4.06)	4.2(7.1)	2.38	0.13	5.3(14.1)	11.7(19.4)	26.14	**<0.0001** *	10.63	**0.003** *
Feet	2.4(3.8)	5.3(9.5)	4.64	**0.04** *	3.5(7.9)	9.8(18.5)	6.59	**0.02** *	0.19	0.66

Note. Mean and standard deviation unit is mm. F value indicates Friedman’s F. Bold font and

* indicate significant values, α = 0.05.

## Discussion

Our study evaluated leg muscle activity, discomfort, and whole-body kinematics during 10 minutes of carrying and moving a load with and without the CarrySuit^Ⓡ^. The results showed that with the exoskeleton, leg muscle activation did not vary with time during the task, and discomfort in the knees, lower leg, and ankles did not increase with time either. Conversely, carrying without the exoskeleton led to some changes in muscle activation over time, and discomfort increased with time in the evaluated leg areas. Regarding kinematics, an increase in ranges of motion over time was observed for both conditions, mainly in sagittal plane, while difference between conditions, regardless of time, were present more in the frontal and transverse planes.

### Leg muscle activity and discomfort

The lack of change in the static and dynamic muscle activation over the 10-minute carrying task, along with no significant discomfort reported in the knees, lower legs, and ankles, may support the use of the tested passive exoskeleton, as there are no signs of muscle fatigue present. In contrast, without the exoskeleton, the increase in discomfort in the evaluated leg areas and the increase in static activation (P_10_) of the vastus lateralis toward the end of task may indicate less opportunity for the muscle relaxation [25]. However, these changes in muscular activation were observed only for females. Overall, our data indicates no clear changes in leg muscle activity, which aligns with similar studies. Indeed, Baltrusch et al. reported no significant changes in leg muscle activity with and without a passive trunk exoskeleton during 5 minutes of walking [13]. Furthermore, the significant increase in discomfort in the hip/upper legs and feet in both conditions seems to be particular to the task and not due to the exoskeleton.

### Whole-body kinematics

#### Sagittal plane ROM

An increase of ROM over the 10 minutes of the carrying task was observed with the exoskeleton, but not without the exoskeleton, for the right ankle, and pelvis for both sexes, and knees for males only. This increase in ROM during the task may correspond to a progressive adaptation to walking with the exoskeleton and suspended load, which is common in motor learning [39]. Although a familiarization period was provided before the experimental task, adaptation may be related to learning the extent to which the exoskeleton supports the carrying task, resulting in a less stiff posture during gait. Without the exoskeleton, the ROM increase over time for both sexes was only observed for the left and right hip. These changes in ROM observed during the carrying task may be due to fatigue of the upper arms, which produced a change in how the load was carried, moving it closer to the body [10]. A review and meta-analysis study reported an increase in hip sagittal plane ROM during backpack carriage while walking [40]. This situation presents some similarity with the CarrySuit^Ⓡ^ tested here, as the latter is worn like a backpack. Hence, it can be inferred that no change over time in right hip flexion-extension ROM with the exoskeleton is an indicator of a positive outcome, as these hip movements are not modified. This is also supported by the lack of change in muscle activation of the vastus lateralis shown above.

Differences between conditions for ROM in the sagittal plane, regardless of time, were found for the elbows, thorax, and neck. When carrying without the exoskeleton, the high ROM of the right and left elbow is expected as the arms are used to lift the box and carry it. However, when the load is suspended to the exoskeleton, the arms can remain in a more relaxed posture to only control lateral movements of the load. This agrees with a previous result showing fewer upper limb muscle activation changes over time with than without the exoskeleton [10]. In females, the greater thorax posterior-anterior tilt ROM with the exoskeleton is also congruent with higher upper body flexibility/less restriction, since low back muscle activity reported previously for this condition is lower [10]. Thus, showing greater benefits for females than males. The lower neck flexion-extension ROM for males only in the EXO condition is not clear since no changes in the thorax were observed between conditions. However, this effect may be associated with neck ROM changes in the transverse plane.

#### Frontal plane ROM

The increase of ROM observed for the ankles over time seems to be relevant to the carrying task, as the changes are similar for both conditions. The effort of walking for 10 minutes while carrying a load may affect this movement pattern and could also be explained by the discomfort in the feet. Changes in ankle biomechanics have been previously observed when carrying and walking with heavy loads relative to unloaded walking [41]. However, the increase in right elbow adduction-abduction ROM may be associated with the lateral control of the load during the carrying task, as the box is suspended to the exoskeleton, and the large majority of the participants were right-hand dominant (Fig 1).

Differences between conditions for the frontal plane ROM, regardless of time, were found for the right knee, right hip, and thorax. Similar to the changes in ROM observed in the sagittal plane, the thorax ipsi-contra lateral tilt ROM of the females was greater with the exoskeleton. This corroborates the benefits mentioned above, which align with the lower erector spinae activation as reported earlier [10]. The exoskeleton also allowed for greater adduction-abduction ROM for the right hip for both sexes; this seems to be a positive outcome since trunk loading reduces hip abduction/adduction. Similar results were observed in a study with police officers carrying a load-bearing vest and a safety vest [42]. For the right knee, a lower adduction-abduction ROM was observed only in females with the exoskeleton, which could be associated with the larger variability in female knee kinematics when carrying heavy loads [43].

#### Transverse plane ROM

No changes over time were observed for ROM in the transverse plane; however, a greater ROM of the hips and thorax was observed for females with the exoskeleton. This thorax flexibility also agrees with the lower erector spinae activation observed with the exoskeleton in our previous paper [10], indicating a more natural posture of the torso in all three planes of movement than without the exoskeleton. In studies where a load is fixed to the trunk, reduced rotational amplitudes of the trunk have commonly been observed, as reported in police offices with heavy vests [42], healthy subjects with spinal braces [44], and backpack carriage [45–47]. For the males, thorax ipsi-contra lateral rotation ROM was lower with the exoskeleton, which could be related to the unchanged right erector spinae activation in both conditions [10], showing again more benefits of the exoskeleton for females. The greater internal-external rotation ROM of the hips could be related to the adaptations needed for controlling the frontal carriage and swaying of the load hanging from the exoskeleton. Alternatively, the lower position of the load due shorter stature of the females may interfere with the upper leg movement an induced more internal-external rotation ROM (Fig 1). For males, the greater neck axial rotation ROM without the exoskeleton may result from intrinsic head movement activity while carrying a load, since the change is similar to flexion-extension ROM. In this experiment, males seem to be more head movers than females without the exoskeleton, and this movement is probably limited by the shoulder frame of the exoskeleton.

### Limitations

The laboratory simulation was limited to a duration of 10 minutes, which may not fully capture the long-term effects of exoskeleton usage or the demands of real-world work tasks. Moreover, the familiarization/warm-up period with the exoskeleton was limited to one minute, adaptation effects should be considered in future studies. The findings should be interpreted with caution as they may not fully reflect the complexities and variations of actual work conditions. The study acknowledged that exoskeletons are designed for specific purposes and may not cover all tasks performed in real manual material handling jobs, such as lifting and lowering. The findings should be interpreted within the context of the specific exoskeleton used in this study, and the results may not be generalizable to other exoskeleton designs or configurations. For EMG data, the Mean-Task method was chosen to normalize muscle activity, but alternative normalization methods could be employed to directly compare muscle work between conditions. The walking space for the carrying task and the specific measurement intervals for assessing ROM allowed only one complete gait cycle of the right leg per measurement period. Using longer recordings could provide a more comprehensive understanding of kinematics and symmetry. The study sample consisted of a specific population of young healthy individuals and may not represent the wider range of individuals who use exoskeletons or engage in carrying activities. Thus, it is necessary to investigate the impact of the CarrySuit^Ⓡ^ in other populations with different characteristics, such as age, physical fitness, or occupation.

## Conclusions

This study provides insights into the effects of a passive exoskeleton usage on the range of motion in various joints, leg muscle activity and discomfort during a carrying task. The results indicate that leg muscle activation did not change over time with the use of the exoskeleton. In this condition, discomfort in the knees, lower leg, and ankles was not significantly affected by time. This suggests that the tested exoskeleton may be utilized without having a major impact on leg muscle fatigue. In contrast, performing the task without the exoskeleton led to an increase in discomfort over time and changes in muscle activation, particularly in females. The results also indicate that exoskeletons can influence joint motion and potentially reduce musculoskeletal strain. The higher ROM in certain joints suggests greater benefits for females than males. However, the effectiveness and usability of exoskeletons may vary depending on the specific joint and sex. Overall, based on the objective and subjective measurements evaluated in this study, the CarrySuit^Ⓡ^ exoskeleton seems to be a suitable aid for users during a carrying task. The long-term effects remain to be investigated to optimize exoskeletons design, and the effects of exoskeletons in different occupational settings and populations need further attention.

## Supporting information

S1 Fig (PDF)

S2 Fig (PDF)

S3 Fig (PDF)

S4 Fig (PDF)
