# Supplementary figures and images for: Effects of a passive upper-body exoskeleton on whole-body kinematics, leg muscle activity, and discomfort during a carrying task
Source: PLoS One. 2024 Jul 11;19(7):e0304606. doi: 10.1371/journal.pone.0304606 (PMC11238980; doi:10.1371/journal.pone.0304606)

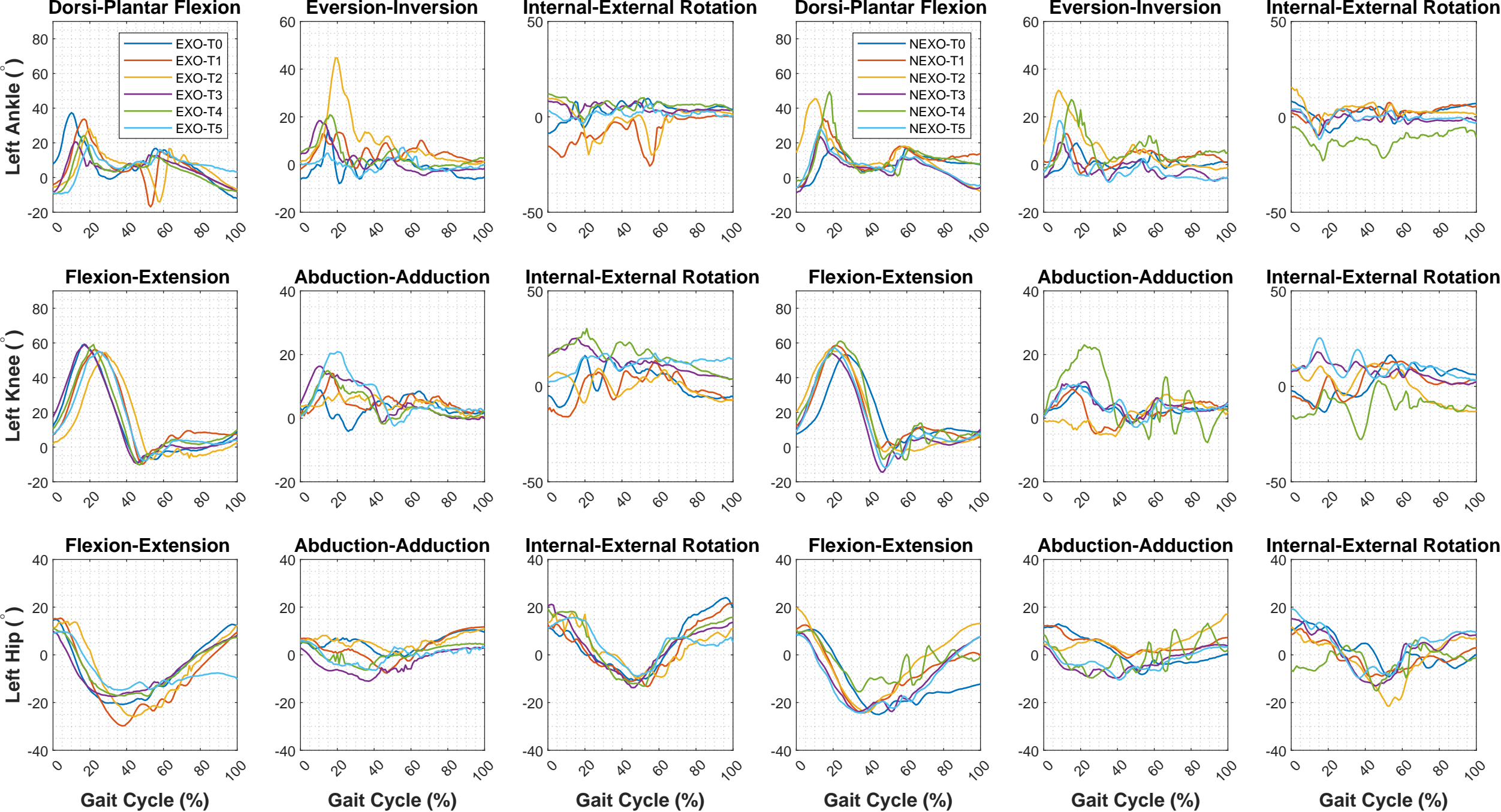

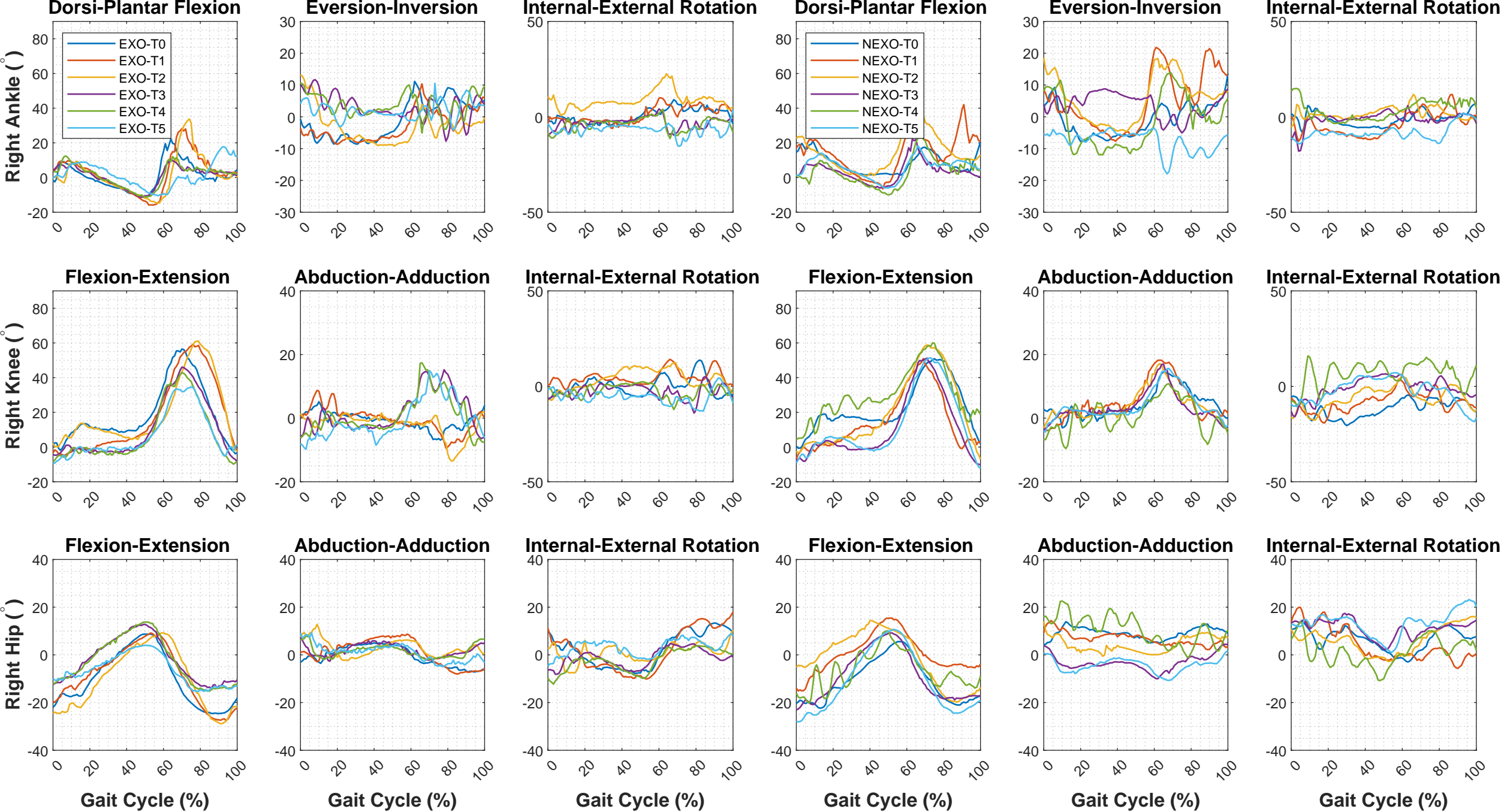

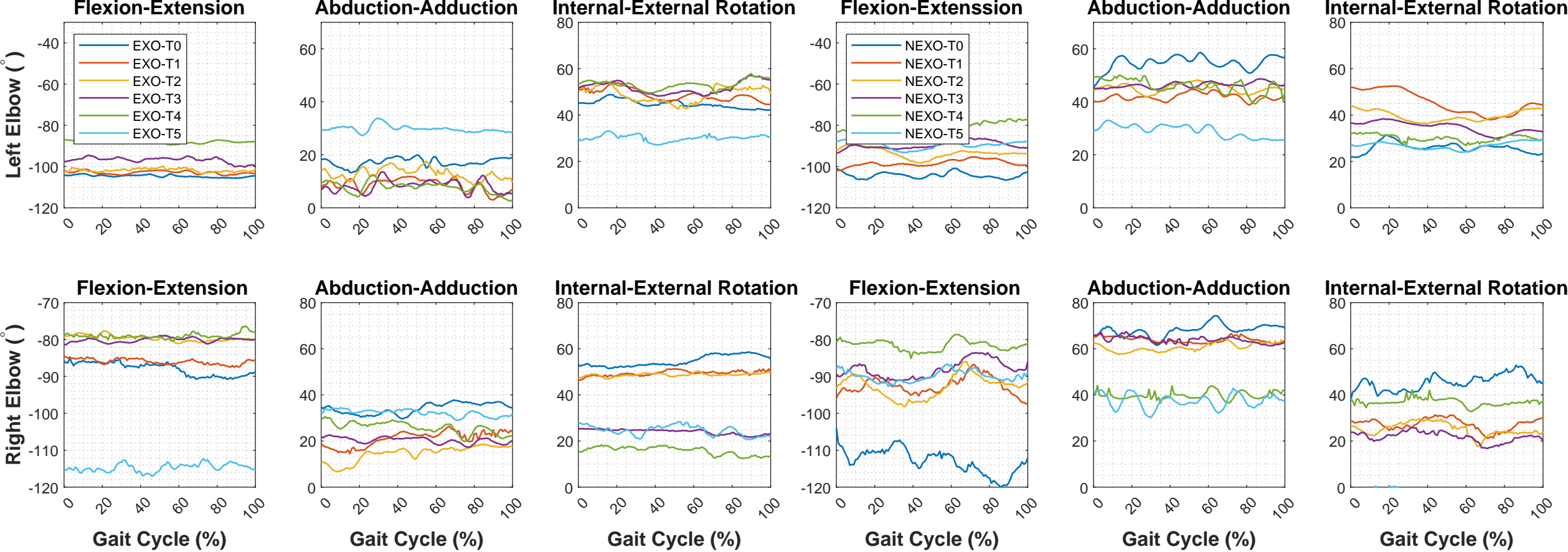

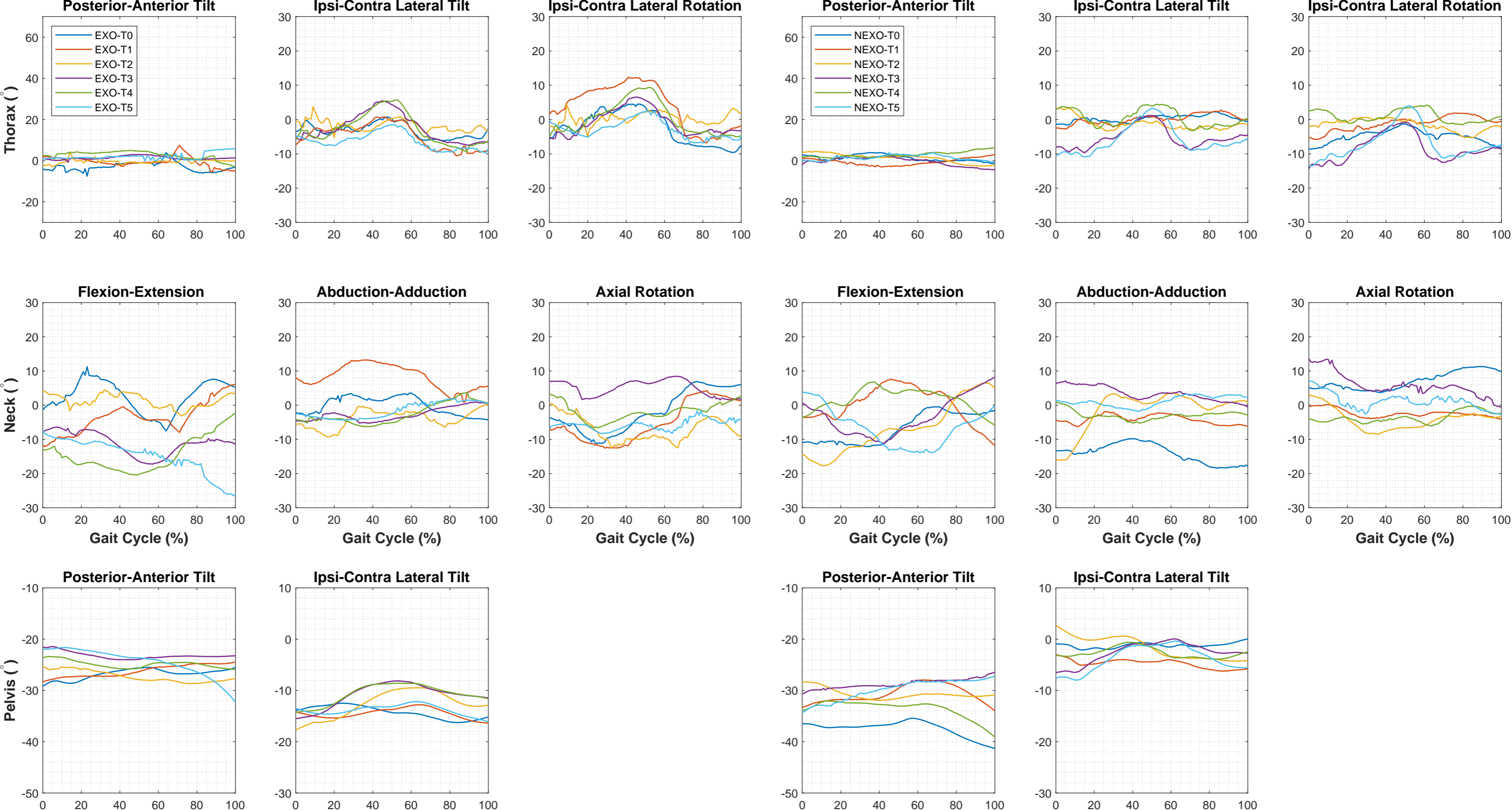

Supplement: S1 Fig — (PDF) [file pone.0304606.s001.pdf]

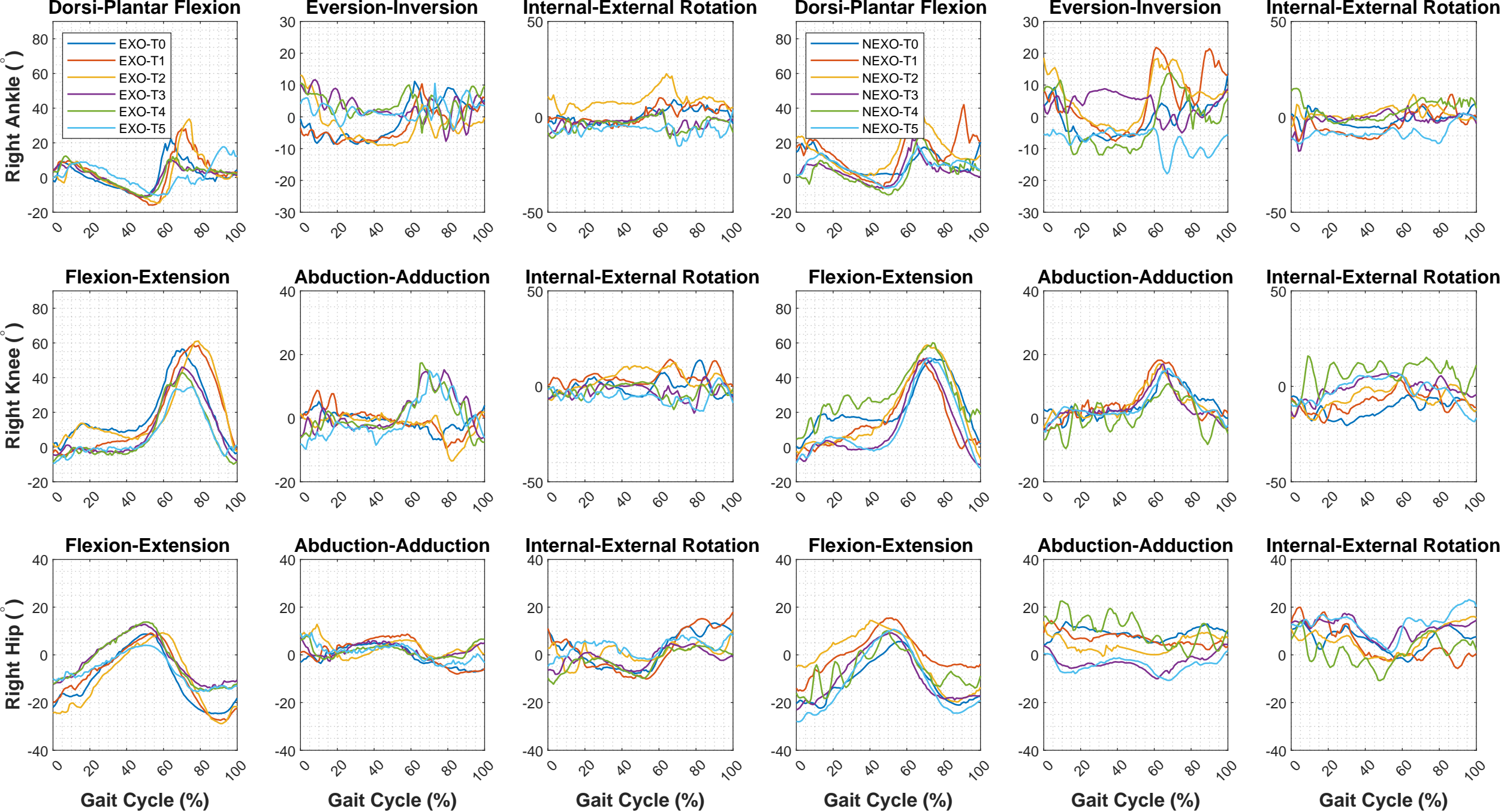

Supplement: S2 Fig — (PDF) [file pone.0304606.s002.pdf]

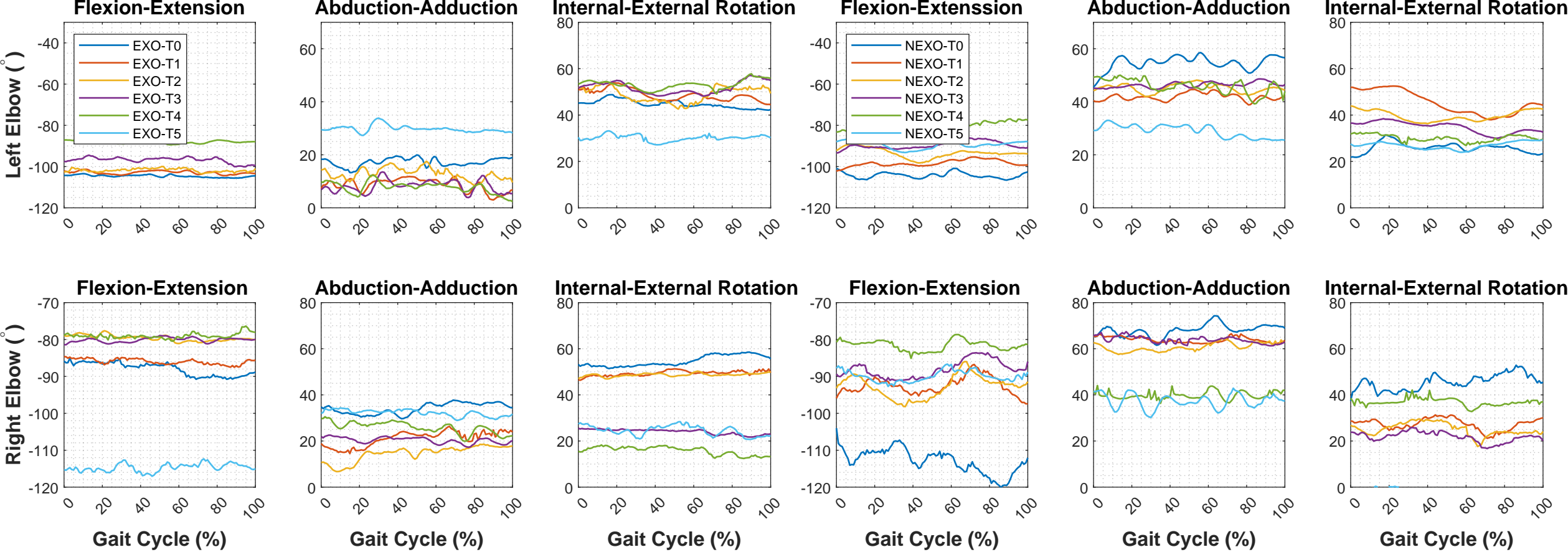

Supplement: S3 Fig — (PDF) [file pone.0304606.s003.pdf]

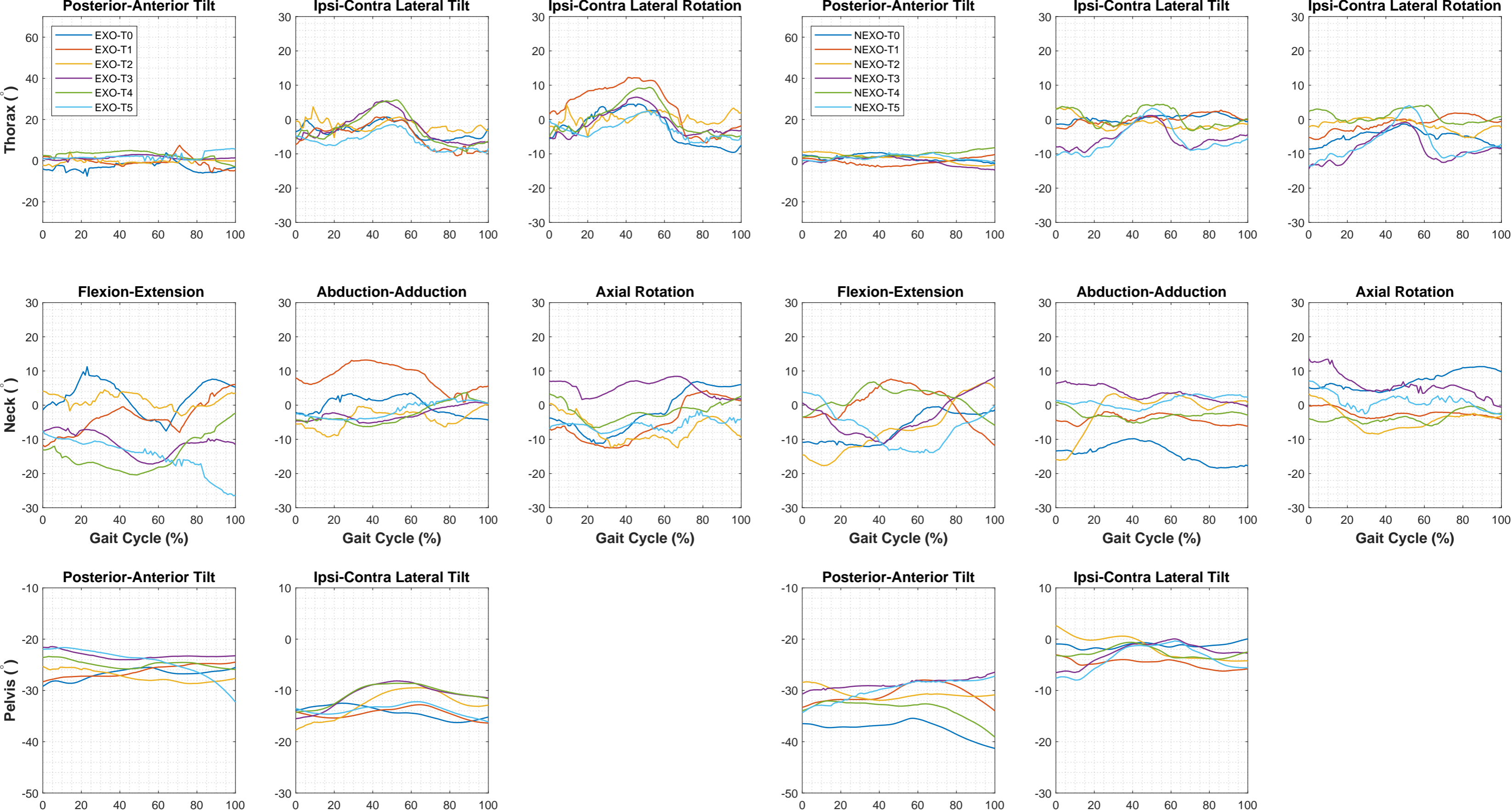

Supplement: S4 Fig — (PDF) [file pone.0304606.s004.pdf]
